# Repeated extrinsic rewards following retrieval practice facilitate later memory

**DOI:** 10.3758/s13423-026-02860-4

**Published:** 2026-03-31

**Authors:** Devyn E. Smith, Amanda M. Smith, Hannah R. Buras, Nicole M. Long

**Affiliations:** Department of Psychology, University of Virginia, PO Box 400400, Charlottesville, VA 22904 USA

**Keywords:** Memory, Retrieval, Reward, Practice

## Abstract

The anticipation of extrinsic reward facilitates memory formation. However, it is unclear how reward following memory retrieval influences the information that is retrieved and later remembered. Here, we conducted four behavioral experiments in which we manipulated retrieval practice reward delivery. Across all experiments, participants studied word-image pairs and then completed two rounds of retrieval practice, followed by a final recognition test. Participants made vividness judgments during retrieval practice, and in three of four experiments, each response had a 50% chance of yielding positive feedback. We find that repeated rewards following retrieval practice facilitate later memory. Items that are practiced with low vividness are remembered worse than items that are not practiced. Together, these results suggest that the benefits of both retrieval practice and reward may depend on the strength of the memory retrieved.

## Introduction

Episodic memories may be reinforceable through extrinsic rewards (e.g., monetary compensation) as information that is valuable or rewarding is prioritized over information that is less rewarding (Dickerson & Adcock, 2018; Loftus & Wickens, 1970). The potential for future rewards enhances memory formation or encoding (Adcock, Thangavel, Whitfield-Gabrieli, Knutson, & Gabrieli, 2006). However, the extent to which reward *following* memory retrieval impacts the contents of retrieval and subsequent behavior is unknown. Prior behavioral work that has investigated test-phase extrinsic reward has found mixed results (Castanheira, Lalla, Ocampo, Otto, & Sheldon, 2022; Shigemune, Tsukiura, Nouchi, Kambara, & Kawashima, 2017). However, these studies used anticipatory methods, which, like studies employing value-directed remembering (Castel, 2007; Castel, Benjamin, Craik, & Watkins, 2002), have the potential to modulate the strategies that individuals use, rather than modulating the contents of retrieval. Thus, it remains an open question how receiving an unexpected reward immediately after memory retrieval affects processing and subsequent behavior. Memory reinforcement may be better accomplished through direct reward of what is retrieved, rather than through manipulation of potential future reward, and may more closely mirror intrinsic responses to retrieval success (Smith & Long, 2024; Speer, Bhanji, & Delgado, 2014). The aim of this study was to investigate how extrinsic reward following memory retrieval impacts subsequent memory.

Associating a study item with a potential reward – e.g., reward that will be received if the item is remembered at test – impacts the likelihood that an item is later remembered (Elliott, Blais, McClure, & Brewer, 2020; Loftus & Wickens, 1970; Marini, Marzi, & Viggiano, 2011). Higher potential rewards lead to better subsequent memory and drive correlated activity between reward regions (e.g., ventral tegmental area, striatum) and the hippocampus. Reward signals upregulate hippocampal memory encoding mechanisms (Adcock et al., 2006; Wolosin, Zeithamova, & Preston, 2012). Given that the hippocampus supports both memory encoding and memory retrieval (Diana, Yonelinas, & Ranganath, 2007; Eichenbaum, 2004; Long et al., 2017), a similar interaction between the reward system and hippocampus during retrieval may also serve to enhance memory performance.

A limitation of existing studies – regardless of whether potential reward is manipulated during the study or test phase – is that reward delivery is always anticipatory. That is, individuals are aware prior to encountering a study or test stimulus that there is the potential to receive a reward for remembering that stimulus. In value-directed remembering paradigms, participants can selectively attend to high-value information and apply elaborative encoding strategies to high-value items (Castel, 2007; Castel et al., 2002; Filiz & Dobbins, 2024; Knowlton & Castel, 2022). Thus, the discrepancy in the existing studies employing test-phase rewards (Castanheira et al., 2022; Shigemune et al., 2017) may be driven by differences in strategy adoption, specifically how participants choose to attempt to retrieve. For instance, in the presence of a potential high-value reward, participants may exert increased retrieval effort (Rugg & Wilding, 2000), altering how the cue stimulus is processed. In contrast, non-anticipated rewards following retrieval are unlikely to consistently modify *anticipatory* strategy engagement. Random rewards during study are thought to promote dopaminergic consolidation processes (Murayama & Kitagami, 2014). Post-retrieval reward may similarly lead to alterations in memory representations – rather than retrieval itself – and how the contents of retrieval are processed. Thus, it is important to identify how an extrinsic reward immediately following memory retrieval affects the information retrieved.

Understanding the impact of post-retrieval reward is important because rewards can be both facilitatory and deleterious. High-value rewards can have negative effects on memory (Chung & Federmeier, 2023). Although retrieval practice is known to facilitate memory (Karpicke, 2012; Roediger & Karpicke, 2006), partial retrieval has the potential to negatively impact later memory. According to the non-monotonic plasticity hypothesis, a memory representation is weakened when it is moderately or partially reactivated (Poppenk & Norman, 2014). Thus, how vividly a memory is reactivated during retrieval practice will impact its later memory (Kuhl, Johnson, & Chun, 2013; Lee, Samide, Richter, & Kuhl, 2018). Furthermore, retrieval practice can increase errors to similar novel stimuli if general information common to both study items and lures is strengthened (Lee et al., 2018; McDermott, 2006). That is, retrieving gist-level category information is likely to increase both true and false memory (Brainerd & Reyna, 2002). Presenting rewards during retrieval practice may or may not serve to facilitate later memory. Rewards administered following retrieval may serve to upregulate attention to the reactivated representation, strengthening a weak or low-vivid representation that would otherwise be forgotten. Alternatively, as rewards can extend across related items (Oyarzún, Packard, de Diego-Balaguer, & Fuentemilla, 2016) and to inferred associations (Wimmer & Shohamy, 2012), rewards may promote false memories. To the extent that participants reactivate category-level information during retrieval practice (e.g., Lee et al., 2018), rewards could reinforce gist-like representations over item-specific or verbatim representations, which could lead to an increase in false memories for categorically related lures presented during test.

Our hypothesis is that extrinsic reward following retrieval will reinforce the information that is retrieved and modulate subsequent memory. To test our hypothesis, we conducted four behavioral experiments in which we manipulated test-phase reward delivery. Across all experiments, participants studied word–image pairs and then completed two rounds of retrieval practice, followed by a final recognition test. During retrieval practice, participants were given a word cue and instructed to bring to mind the associated image. They rated the vividness of their memory for the image on a scale from one (least vivid) to four (most vivid). In Experiments 1–3, every response had a 50% chance of receiving reward feedback. We separately investigated the impact of reward on hit and false-alarm rates, anticipating that contributions of item- vs. category-level information would differentially affect true and false memory. To the extent that reward reinforces the information retrieved, we should find higher hit rates for rewarded items than for not rewarded items.

## Materials and methods

### Participants

A total of 168 native English speakers participated, with 42 participants enrolled in each experiment (E1: 22 female; age range = 18–22, mean age = 19.2 years; E2: 27 female; age range = 18–21, mean age = 18.8 years; E3: 26 female; age range = 18–21, mean age = 19 years; E4: 25 female; age range = 18–21, mean age = 19.1 years). All participants had normal or corrected-to-normal vision. Informed consent was obtained in accordance with the Institutional Review Board for Social and Behavioral Research and participants received class credit for their participation. Our sample size was determined a priori based on pilot data (E2, N = 14) described in the pre-registration report of this study (osf.io/gebm4). A total of 30 participants were excluded from the final dataset. Two (one each from E2 and E4) were excluded due to having a d’ > 2.5 SDs of the mean across the four experiments. Twenty-eight participants (E1: 8; E2: 5; E3: 10; E4: 5) were excluded due to failing to respond to all interleaved odd/even trials (see below) during the study phase. Thus, data are reported for the remaining 138 (E1: 34, E2: 36, E3: 32, E4: 36) participants. All raw, de-identified data and the associated experimental and analysis codes used in this study are available via the Open Science Foundation (osf.io/y5ac8).

**Fig. 1:**
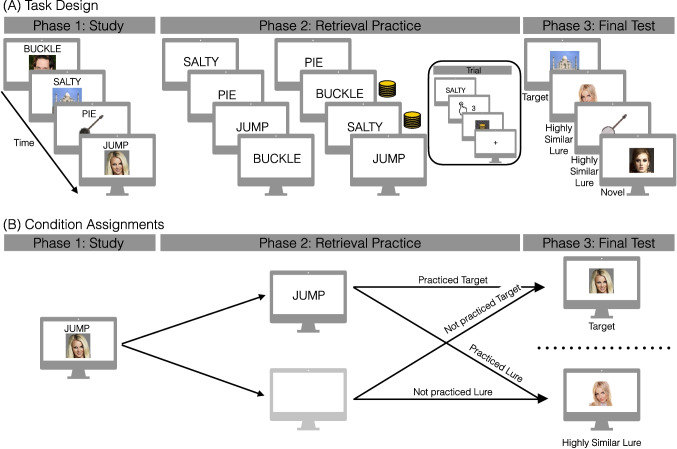
Task design and condition assignments. **A** During phase 1, participants studied word–image pairs; images were from one of three categories: famous faces (e.g., Britney Spears), famous scenes (e.g., Taj Mahal), and common objects (e.g., banjo). In phase 2, participants completed two rounds of retrieval practice. Participants saw an individual word and were instructed to bring to mind the image associated with each word and make vividness ratings on a scale from 1 to 4, with 1 being least vivid and 4 being most vivid. In E1, during the first round of retrieval practice, every response had a 50% chance of receiving reward feedback displayed as coins over a mask. The mask is shown alone on no-reward trials. In E2, during the second round of retrieval practice, every response had a 50% chance of receiving a reward. In E3, participants received rewards during both rounds of retrieval practice. In the first round, as in E1, every response had a 50% chance of receiving a reward. If a reward followed an item in the first round of retrieval practice, a reward followed the same item in the second round. In E4, participants did not receive any reward during either round of retrieval practice. The temporal dynamics of a trial during retrieval practice are as follows: word is presented, participants rate the vividness of their memory for the image, then a reward could immediately follow. All participants then completed phase 3, a final recognition memory test that included images only. Test probes included previously studied images (targets), highly similar lures (non-identical images depicting the same person, place, or object as the targets), and novel images. Participants made old or new judgments using a confidence rating scale from 1 to 4, with 1 being definitely new and 4 being definitely old. **B** We refer to both targets and lures as ‘practiced’ or ‘not practiced’ on the basis of whether the association (e.g., JUMP-Britney Spears) was practiced or not (greyed monitor image) during phase 2. Participants would only ever experience one condition per association, meaning that no participant would see both images of Britney Spears. These conditions can be further subdivided on the basis of rewards and/or vividness rating during phase 2 (see Fig. 2)

**Fig. 2:**
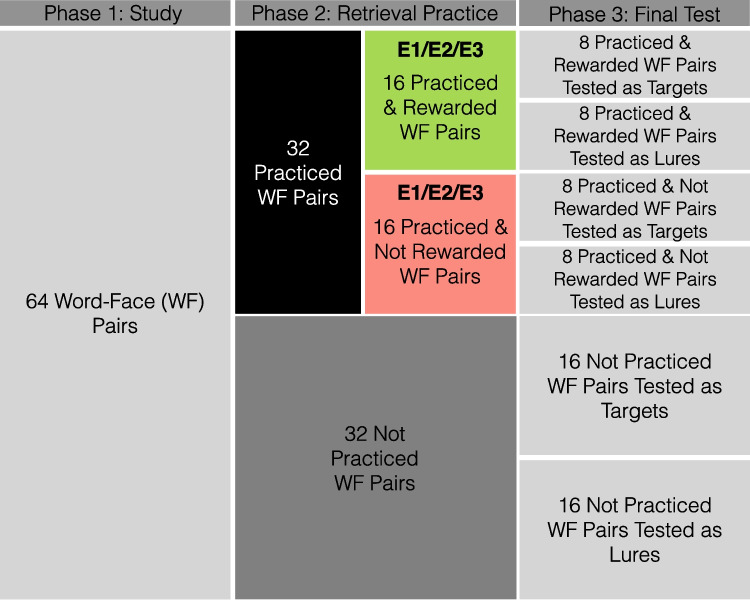
Condition divisions and counts. For each image category, 64 word–image pairs are shown during the phase 1 study. We show the specific example of word–face (WF) pairs here, but the same applies to word–object and word–scene pairs. Across all four studies, of the 64 studied WF pairs, 32 are practiced and 32 are not practiced. For E1, E2, and E3, the 32 practiced pairs are further sub divided into 16 rewarded and 16 not rewarded practiced pairs. During the phase 3 final test, pairs are tested as either targets (the exact image presented during study is presented during test) or lures (non-identical images depicting the same face as the phase 1 image). Therefore, across all four experiments there are 16 not practiced pairs tested as targets and 16 not practiced pairs tested as lures. In E4 which has no rewards there are likewise 16 practiced pairs tested as targets/lures. In E1, E2, E3, there is further sub-division on the basis of reward yielding eight pairs per bin (rewarded/not rewarded and tested as target/lure)

### Recognition task experimental design

We conducted four recognition memory experiments (E1, E2, E3, E4) each with three phases (Fig. 1) and manipulated test-phase reward delivery between participants. Stimuli consisted of 1602 words drawn from the Toronto Noun Pool (Friendly, Franklin, Hoffman, & Rubin, 1982) and three categories of images: 490 common objects (e.g., banjo), drawn from an image database with multiple exemplars per object category (Konkle, Brady, Alvarez, & Oliva, 2010), 96 famous faces (e.g., Britney Spears) and 96 famous scenes (e.g., Taj Mahal; Lee et al., 2018). From this set, 192 words and 288 images were selected for each participant. The images consisted of an equal number (96) of objects, faces, and scenes. Of the 288 images, a subset of 192 was presented in phase 1, with 64 images drawn from each visual category. Trial counts by condition are shown in Fig. 2. Only one exemplar per object category appeared during phase 1 (e.g., one banjo). Word–image associations were randomly generated for each participant and randomly assigned to condition (e.g., target or lure, see below).

*Phase 1: Study.* In each of four runs, participants studied 48 word–image pairs, yielding a total of 192 trials. On each trial, participants saw a word–image pair presented for 2000 ms followed by a 3500-ms distractor interval. The distractor interval was comprised of alternating fixation and digit presentation (fixation, digit, fixation, digit, fixation). During digit presentation, participants saw a single digit, 1 through 10, and were instructed to press one of two buttons (“1” or “2”) to indicate if the number was odd or even. Each fixation was 500 ms and each digit presentation was 1000 ms.

*Phase 2: Retrieval practice.* Participants completed two rounds of retrieval practice containing 96 trials each. A total of 96 words from phase 1 were presented. The same words were presented in both rounds of retrieval practice in random order. Each round was further subdivided into two runs with 48 trials. In each run, an equal number of words (16) associated with an image from each visual category were presented. On each trial, participants were presented with a word cue for 4000 ms and instructed to bring to mind the associated image from the study phase. Participants rated the vividness of their memory of the retrieved image on a scale from 1 to 4, with 1 being least vivid and 4 being most vivid. To motivate participants to use the full response scale, if the same vividness response was made on ten consecutive trials, participants received a message instructing them to use the full scale. Thirty-one of 138 participants saw the message at least once; the proportion of participants who saw the message did not differ across experiments (E1: 12/34, E2: 7/36, E3: 5/32, E4: 7/36; $\chi {^2}$ = 2.40, *p* = 0.12). Following the word cue, participants saw either a scrambled mask or feedback displayed as coins overlaid on the scrambled mask for 1000 ms, followed by a 500-ms inter-stimulus interval (ISI).

We manipulated the schedule of reward delivery across experiments to test how reward structure – when and how many rewards were presented – impacts subsequent memory. We varied whether rewards were presented in either the first, second, both, or neither rounds of retrieval practice. We expected that repeated rewards would have the largest positive impact on subsequent memory and that rewards presented exclusively in the first round of retrieval practice would negatively impact subsequent memory. In E1, participants received rewards during only the first round of retrieval practice. Every response had a 50% chance of receiving reward feedback. In E2, participants received rewards during only the second round of retrieval practice. As in E1, every response had a 50% chance of receiving reward feedback. In E3, participants received rewards during both rounds of retrieval practice. In the first round, as in E1, every response had a 50% chance of receiving reward feedback. If a reward followed an item in the first round of retrieval practice, a reward followed the same item in the second round. In E1, E2, and E3, 48 total words were rewarded (16 words associated with images from each visual category). In E4, participants did not receive any reward during either round of retrieval practice.

*Phase 3: Final test.* Participants completed a final recognition memory test for images only. Trials were self-paced, and participants made old or new judgments for each image using a confidence rating scale from 1 to 4, with 1 being definitely new and 4 being definitely old. Trials were separated by a 500-ms ISI. There were a total of 288 test trials. Test probes included 96 previously studied images (targets), 96 highly similar lures (non-identical images depicting the same face, scene, or object as the phase 1 image), and 96 novel face, scene, or object images. To reduce test-phase interference, participants were only tested on either a target (e.g., original image of Britney Spears) or the similar lure (e.g., new image of Britney Spears), as in prior work (Lee et al., 2018). There were an equal number of images from each visual category for each test probe condition (i.e., 32 novel scenes). Half (48) of the targets were practiced and half were not practiced; likewise, half of the lures were associated with an image that was practiced and half were associated with an image that was not practiced. We refer to these as “practiced lures” and “not practiced lures” although the lures themselves were not practiced (Fig. 1B). For E1, E2, and E3, half (24) of the practiced targets were rewarded during retrieval practice and half were not rewarded. Similarly, half of the practiced lures were associated with an image that was rewarded during retrieval practice and half were associated with an image that was not rewarded during retrieval practice. We refer to these as “rewarded targets” and “rewarded lures” although the rewards were always presented during the phase 2 retrieval practice and never during the final phase 3 recognition test.

**Table 1:** Hit and false-alarm rates as a function of practice, reward, and experiment

	Hits			FAs			
	Practiced, Rewarded	Practiced, Not Rewarded	Not Practiced	Practiced, Rewarded	Practiced, Not Rewarded	Not Practiced	Novel Items
	Mean (SD)	Mean (SD)	Mean (SD)	Mean (SD)	Mean (SD)	Mean (SD)	Mean (SD)
E1	0.5882 (0.1613)	0.614 (0.1567)	0.6036 (0.1285)	0.3382 (0.145)	0.3444 (0.1517)	0.3272 (0.1316)	0.1762 (0.1297)
E2	0.603 (0.1576)	0.5984 (0.1361)	0.5978 (0.1532)	0.3565 (0.1404)	0.3553 (0.137)	0.3681 (0.1118)	0.1539 (0.0915)
E3	0.651 (0.1408)	0.5846 (0.1803)	0.6146 (0.1566)	0.3268 (0.1413)	0.3346 (0.1328)	0.3392 (0.1108)	0.1169 (0.078)
E4	−	0.5972 (0.1408)	0.5874 (0.1291)	−	0.3356 (0.1162)	0.353 (0.1152)	0.127 (0.0706)

**Fig. 3:**
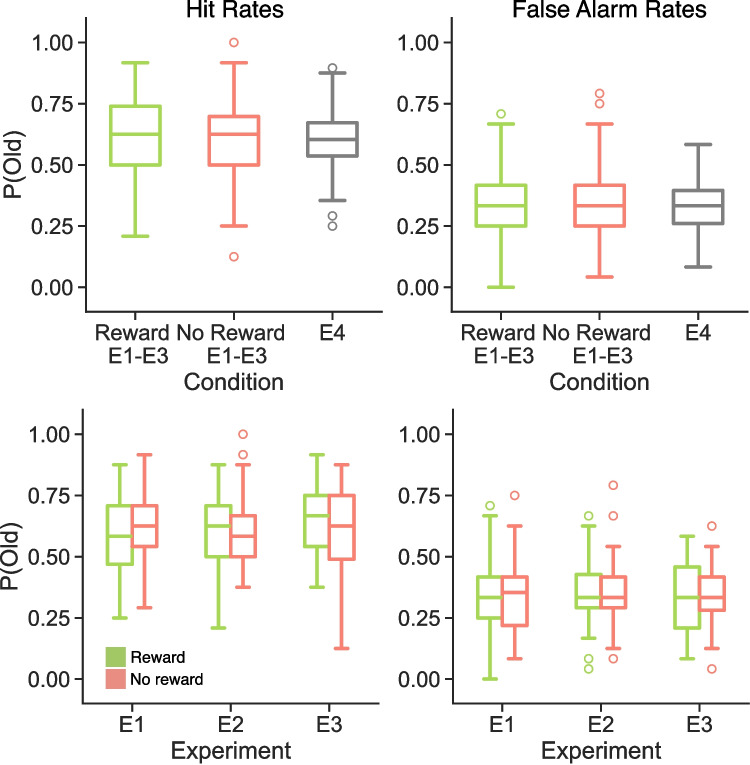
Influence of reward delivery and reward structure on hit and false-alarm rates. Each panel shows the proportion of old responses to reward (*green*), no reward (*red*), and/or E4 (*grey*) probes. The *left columns* show hit rates and the *right columns* show false-alarm rates. The *top row* shows data averaged over experiments 1–3; the *bottom row* shows data separately for each experiment. Box-and-whisker plots show median (*center line*), upper and lower quartiles (*box limits*), 1.5$\times$ interquartile range (*whiskers*) and outliers (*circles*). We do not find a significant difference in either hit rates or false-alarm rates for reward or no reward E1-E3 probes compared to E4 probes. We find a significant interaction between reward and experiment (*p* = 0.0385) on hit rates, but not on false-alarm rates (*p* = 0.9505)

#### Statistical analyses

We used mixed-effects ANOVAs (meANOVAs) to assess the effects of retrieval practice and reward structure on hit rate and false-alarm (FA) rate. “Hits” were defined as any target that received a response of “old,” regardless of confidence. “False alarms” were defined as any lure that received a response of “old,” regardless of confidence. We collapsed over confidence to maximize the number of trials per condition. Consistent with our pre-registration as well as past work (Lee et al., 2018), we excluded novel items. Additionally, we excluded novel items because they did not intersect with our conditions of interest (e.g., practice, reward) and because the proportion of false alarms to novel items was low (Table 1). We used an independent samples *t*-test to compare E1-E3 (rewarded and not rewarded) hit rate and FA rate to E4 hit rate and FA rate. We used repeated-measures ANOVAs (rmANOVAs) to assess the effects of vividness ratings on hit rate and FA rate. We conducted Bayes factor analysis using the BayesFactor package (version 0.9.12-4.7) in R (version 4.4.0) with the default prior settings. We used the functions lmBF and ttestBF to compare models.

**Table 2:** Hit and false-alarm rates for rewarded/not rewarded practice (E1/E2/E3) compared to control (E4)

	Hits	vs. Control				FAs	vs. Control			
	Mean (SD)	t$_{136}$	*p*	*d*	*BF*	Mean (SD)	t$_{136}$	*p*	*d*	*BF*
Rewarded	0.6132 (0.1561)	0.536	0.5929	0.1072	0.2331	0.3411 (0.1428)	0.2046	0.8382	0.0418	0.2089
Not rewarded	0.5993 (0.1583)	0.068	0.9459	0.0136	0.2055	0.3452 (0.141)	0.3616	0.7182	0.0738	0.2174
Control	0.5972 (0.1408)	−	−	−	−	0.3356 (0.1162)	−	−	−	−

Stimulus category – object, face, or scene – had a main effect on recognition performance. Hit rates were significantly higher for objects (M=0.63, SD=0.18) and faces (M=0.63, SD=0.18) compared to scenes (M=0.55, SD=0.19; objects vs. scenes: *t*$_{137}$=3.72, *p*$=$0.0003, *d*$=$0.40, BF=60.41; faces vs. scenes: *t*$_{137}$=5.28, *p*<0.0001, *d*$=$0.43, BF=2.5x$10^{4}$). False-alarm rates were significantly lower for objects (M=0.24, SD=0.15) compared to both faces (M=0.38, SD=0.18; *t*$_{137}$=8.00, *p*<0.0001, *d*$=$0.82, BF=1.5x$10^{10}$) and scenes (M = 0.40, SD=0.18, *t*$_{137}$=9.94, *p*<0.0001, *d*$=$0.96, BF=7.4x$10^{14}$). However, as stimulus category did not interact with any of our conditions of interest, we collapsed over category in all reported analyses.

**Table 3:** Hit rates as a function of reward structure

	E1 vs. E3				E2 vs. E3			
Effect	df	*F*	*p*	$\eta _p^{2}$	df	*F*	*p*	$\eta _p^{2}$
Main effect of reward	(1,64)	0.959	0.3310	0.01	(1,66)	**4**.**105**	**0**.**0468**	**0**.**06**
Main effect of experiment	(1,64)	0.227	0.635	0.003	(1,66)	0.251	0.618	0.003
Interaction of reward $\times$ experiment	(1,64)	**5**.**671**	**0**.**0202**	**0**.**08**	(1,66)	3.436	0.0682	0.05

Note: Bold values indicate $p<$ 0.05

## Results

### Reward structure impacts subsequent hit rates

Our first goal was to test whether rewarded practice, regardless of the structure of reward delivery, modulates memory performance. We report mean and standard deviation of hit and false-alarm rates as a function of practice, reward, and experiment in Table 1. Following our pre-registration, we collapsed data from the three experiments with reward (E1, E2, E3) and compared hit and FA rates from these experiments to hit and FA rates in the control experiment (E4) that had no rewards (Fig. 3). Insofar as extrinsic rewards reinforce the contents of retrieval, we expected to find higher hit rates and lower FA rates for rewarded compared to control trials. However, to the extent that participants retrieve gist or category-level information (Brainerd & Reyna, 2002), we might find higher FA rates for rewarded compared to control trials. We used independent samples *t*-test to compare hit rates and FA rates and find no significant differences across the rewarded and control experiments (Table 2). Thus, we do not find evidence that retrieval practice rewards impact recognition memory performance.

**Fig. 4:**
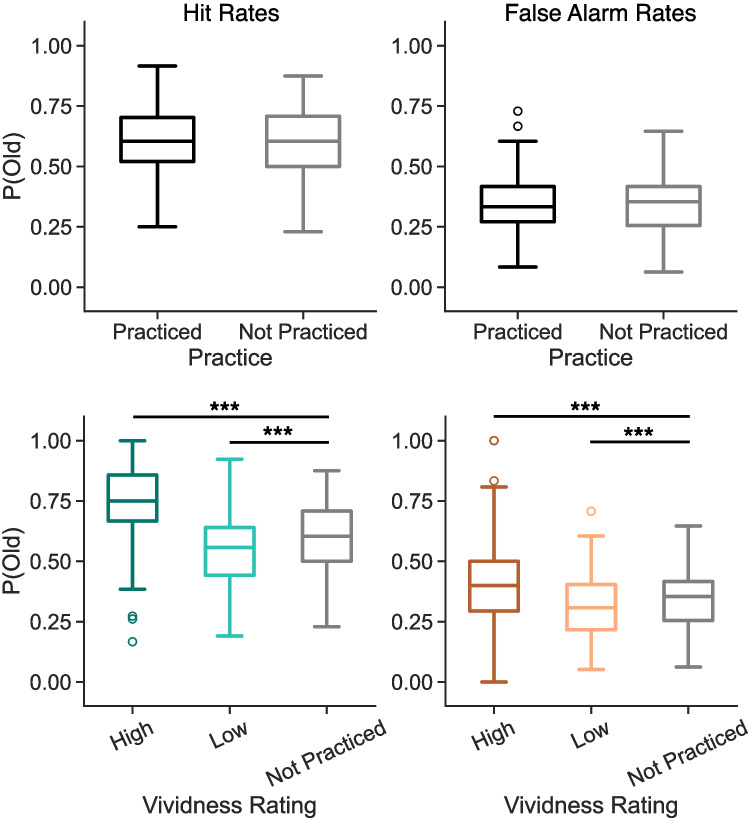
Influence of retrieval practice and vividness on hit and false-alarm rates. Each panel shows the proportion of old responses to probes. Box-and-whisker plots show median (*center line*), upper and lower quartiles (*box limits*), 1.5$\times$ interquartile range (*whiskers*) and outliers (*circles*). **A** We do not find a significant difference in hit rates between practiced (*black*) and not practiced (*gray*) targets (*p* = 0.6535). **B** We do not find a significant difference in false-alarm rates between practiced and not practiced lures (*p* = 0.4015). **C** We find a significant effect of vividness driven by greater hit rates for high-vivid (*dark teal*) compared to not practiced targets and for not practiced compared to the low-vivid (*light teal*) targets. **D** We find a significant main effect of vividness driven by greater false-alarm rates for high-vivid (*dark orange*) compared to not practiced lures and for not practiced compared to the low-vivid (*light orange*) lures

Despite failing to find a general difference between rewarded practice and the control experiment, we expected that reward structure might differentially impact subsequent memory. Specifically, we expected that repeated rewards (E3) might increase subsequent hit rates relative to singly presented rewards (E1, E2). Additionally, we expected that the lack of rewards in the second round of practice (E1) might decrease subsequent hit rates. To directly test the effect of reward structure on subsequent hit rates, we performed a 2$\times$3 meANOVA with reward delivery (rewarded, not rewarded) and experiment (E1, E2, E3) as factors, following our pre-registration (Fig. 3). We do not find a significant main effect of either reward delivery (*F*$_{1,99}$=0.92, *p*$=$0.341, $\eta _p^{2}$=0.009) or experiment (*F*$_{2,99}$=0.16, *p*$=$0.852, $\eta _p^{2}$=0.003). We find a significant reward delivery by experiment interaction (*F*$_{1,99}$=3.37, *p*$=$0.039, $\eta _p^{2}$=0.064). Bayes factor analysis revealed that a model with the interaction term (H$_1$) was preferred to a model without the interaction term (H$_0$, BF$_{10}$ = 1.42, anecdotal evidence for H$_1$). These results suggest that the structure of rewards – how many and when they are received – impacts subsequent memory.

As we anticipated a difference between rewards presented once vs. twice, we conducted two follow-up meANOVAs in which we compared E1 and E2 to E3 (Table 3). For the E1 vs. E3 meANOVA, we find a significant reward by experiment interaction, driven by significantly greater hit rates for rewarded vs. not rewarded targets in E3 (rewarded, M=0.6510, SD=0.1408; not rewarded, M=0.5846, SD=0.1803, *t*$_{31}$=2.51, *p*$=$0.018, *d*$=$0.411, BF=2.755) and numerically lower hit rates for rewarded vs. not rewarded targets in E1 (rewarded, M=0.5882, SD=0.1613; not rewarded, M=0.6140, SD=0.1567, *t*$_{33}$=-0.92, *p*$=$0.366, *d*$=$0.162, BF=0.271). Bayes factor analysis revealed that a model with the interaction term (H$_1$) was preferred to a model without the interaction term (H$_0$, BF$_{10}$ = 2.61, moderate evidence for H$_1$). For the E2 vs. E3 meANOVA, we find a main effect of reward, whereby hit rates are greater for rewarded compared to not rewarded trials. Although numerically hit rates were greater for rewarded vs. not rewarded targets in E2 (rewarded, M=0.6030, SD=0.1576; not rewarded, M=0.5984, SD=0.1361), this difference was not significant (*t*$_{35}$=0.22, *p*$=$0.826, *d*$=$0.031, BF=0.183). Bayes factor analysis revealed that a model with the interaction term (H$_1$) was preferred to a model without the interaction term (H$_0$, BF$_{10}$ = 1.01, anecdotal evidence for H$_1$).

Following our pre-registration, we performed the same 2$\times$3 meANOVA on FA rates (Fig. 3). We do not find a significant main effect of reward delivery (*F*$_{1,99}$=0.11, *p*$=$0.741, $\eta _p^{2}$=0.001) or experiment (*F*$_{2,99}$=0.33, *p*$=$0.723, $\eta _p^{2}$=0.007). We do not find a significant reward delivery by experiment interaction (*F*$_{1,99}$=0.05, *p*$=$0.951, $\eta _p^{2}$=0.001). Bayes factor analysis revealed that a model without the interaction term (H$_0$) was preferred to a model with the interaction term (H$_1$, BF$_{10}$ = 0.0919, strong evidence for H$_0$).

Taken together, these findings suggest that repeated reward during retrieval practice can facilitate later target detection.

### Vividness during retrieval practice differentially impacts subsequent memory

Given the relatively limited impact of rewarded retrieval practice on subsequent memory, we next conducted a series of exploratory analyses in which we investigated the effect of practice and vividness on subsequent memory. The modest effect of rewarded practice may be due to strong effects of either practice itself and/or the vividness experienced during practice.

We first tested the extent to which retrieval practice modulates hit and FA rates (Fig. 4). Based on extensive prior work (e.g. Roediger & Karpicke, 2006), we expected to find higher hit and FA rates for practiced relative to not practiced items. We conducted two paired-samples *t*-tests on data aggregated across all four studies and do not find a significant effect of practice for either hit (practiced, M=0.6039, SD=0.1390; not practiced, M=0.6004, SD=0.1425; *t*$_{137}$=0.45, *p*$=$0.654, *d*$=$0.025, BF=0.105) or FA (practiced, M=0.3412, SD=0.1250; not practiced, M=0.3474, SD=0.1186; *t*$_{137}$=-0.84, *p*$=$0.402, *d*$=$0.051, BF=0.134) rates. Thus, we do not find evidence that practice alone modulates subsequent memory.

We were somewhat surprised by the null effect of retrieval practice on subsequent memory given prior evidence for retrieval practice effects with a similar task design (Lee et al., 2018). However, other work has shown that retrieval practice effects are smaller for recognition relative to recall tasks (e.g., Hogan and Kintsch, 1971), potentially accounting for the lack of practice effects we observe. Additionally, we speculate that the vividness with which participants remembered the associated image may influence the effect of retrieval practice on subsequent memory. Given prior evidence that partial or incomplete reinstatement negatively impacts subsequent memory (Newman & Norman, 2010), practice may have a bidirectional effect: enhancement for high-vivid items and decrement for low-vivid items which, when aggregated, would produce the observed null effect of retrieval practice. To test this possibility, we compared hit and FA rates across three conditions: practiced items that received a high vividness rating, practiced items that received a low vividness rating, and not practiced items (Fig. 4). We specifically used vividness ratings from the first round of retrieval practice across all experiments as these initial ratings could not be modulated by reward or practice itself. We conducted two 1$\times$3 rmANOVAs with three levels: high vivid, low vivid, and not practiced. We expected to find that hit and FA rates would increase for high vivid compared to not practiced items, but would decrease for low vivid compared to not practiced items. We find a significant effect of condition for both hits and FAs (hits: *F*$_{2,274}$=127.10, *p*<0.0001, $\eta _p^{2}=$0.481; FAs: *F*$_{2,272}$=25.79, *p*<0.0001, $\eta _p^{2}=$0.159, *Note: one participant was excluded from the FA analysis for not having any high-vivid FAs*). In both cases, these effects were driven by significantly greater hit and FA rates for high-vivid compared to not practiced items and for not practiced items compared to low-vivid items (Table 4).

**Table 4:** Hit and false-alarm rates as a function of practice vividness

	Hits	vs. Not Practiced				FAs	vs. Not Practiced			
	Mean (SD)	*t*$_{137}$	*p*	*d*	*BF*	Mean (SD)	*t*$_{136}$	*p*	*d*	*BF*
High vivid	0.7349 (0.1785)	**10**.**59**	<**0**.**0001**	**0**.**8328**	1.0 x $10^{18}$	0.4177 (0.1877)	**4**.**741**	<**0**.**0001**	**0**.**4411**	300.5
Low vivid	0.5532 (0.1507)	**−4.608**	<**0**.**0001**	**0**.**3216**	4.7 x $10^{4}$	0.3156 (0.1364)	**−3.819**	**0**.**0002**	**0**.**2583**	77.11
Not practiced	0.6004 (0.1425)	−	−	−	−	0.3485 (0.1182)	−	−	−	−

Note: Bold values indicate $p<$ 0.05

**Fig. 5:**
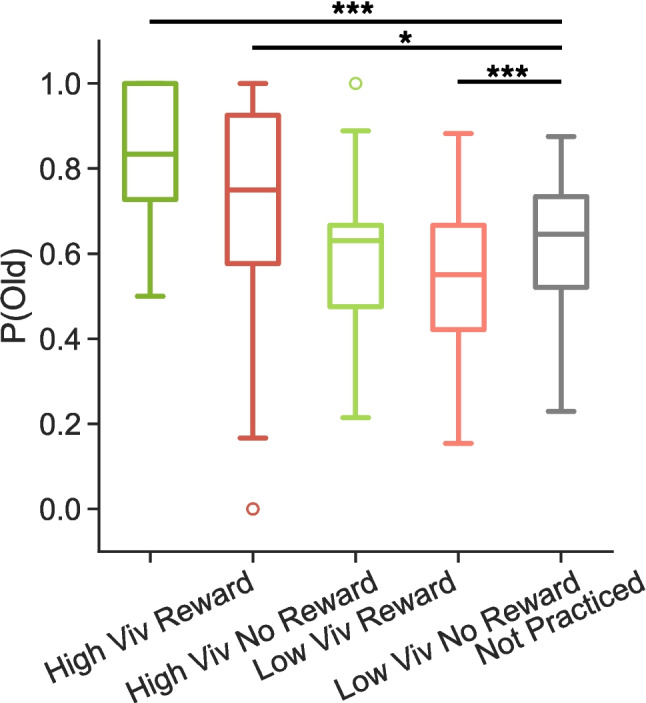
Influence of vividness, reward, and practice on hit rates in E3. We find a significant difference in hit rates between low-vivid targets that were not rewarded and not-practiced targets. Box-and-whisker plots show median (*center line*), upper and lower quartiles (*box limits*), 1.5x interquartile range (*whiskers*), and outliers (*circles*). * *p* < 0.05, *** *p* < 0.0001

### Rewards interact with vividness to modulate subsequent memory

As we have separately shown that repeated rewards (E3) and vividness during practice influence later memory, our final goal was to evaluate the joint impact of rewards and vividness. We focus specifically on hit rates as a function of vividness ratings and reward during the first round of practice in E3, given the significant effect of reward delivery structure on hit rates. We separately consider subsequent hit rates for high- and low-vivid items vs. not practiced items. To the extent that vividness judgments reflect the degree of reinstatement for a given representation (e.g., Kuhl & Chun, 2014), reward following a highly vivid retrieval might have little impact on the associated neural representation, which is presumably already strong or high fidelity. However, to the extent that a low-vivid response during retrieval practice reflects a weak or low fidelity representation, reward may impact that representation and thus later memory.

We performed two 1$\times$3 rmANOVAs to compare high-vivid (rewarded, not rewarded) to not practiced items and low-vivid (rewarded, not rewarded) to not practiced items (Fig. 5) and found a significant effect for each (high vivid: *F*$_{2,62}$=11.81, *p*<0.0001, $\eta _p^{2}=$0.28; low vivid: *F*$_{2,62}$=5.50, *p*$=$0.0006, $\eta _p^{2}=$0.15). We conducted post hoc paired *t*-tests separately for the high- and low-vivid comparisons. We find that hit rates are significantly greater for both high vivid rewarded (M=0.8173, SD=0.1614) and high vivid not rewarded (M=0.6977, SD=0.2846) items compared to not practiced items (rewarded: *t*$_{31}$=7.78, *p*<0.0001, *d*$=$1.28, BF=1.40 $\times$ $10^{6}$; not rewarded: *t*$_{31}$=2.13, *p*$=$0.042, *d*$=$0.362, BF=1.352). We find that hit rates for low-vivid rewarded items (M=0.6016, SD=0.1693) do not significantly differ from hit rates for not practiced items (M=0.6146, SD=0.1566, *t*$_{31}$=-0.48, *p*$=$0.632, *d*$=$0.080, BF=0.211). We find that hit rates for low vivid not rewarded items (M=0.5346, SD=0.1672) are significantly lower than hit rates for not practiced items (*t*$_{31}$=-4.37, *p*$=$0.0001, *d*$=$0.494, BF=200). Together, these results suggest that rewards may ‘amplify’ items that are only partially reinstated or remembered with low vividness.

## Discussion

The aim of this study was to investigate how extrinsic reward following retrieval practice impacts subsequent memory. We conducted four independent recognition memory experiments in which we measured the impact of reward delivery, reward structure, retrieval practice, and vividness on recognition memory. We find that repeated rewards increase subsequent hit rates relative to singly presented rewards and potentially amplify low-fidelity memory representations. Together, these findings suggest that extrinsic rewards following retrieval have the potential to benefit later memory.

We find that reward delivery structure during retrieval practice modulates subsequent memory. Specifically, we find higher hit rates for practice items that were rewarded during both rounds of retrieval practice. As rewards in the current study occurred randomly following the response to the probe, it is unlikely that participants engaged particular strategies *prior* to probe onset as in traditional anticipatory designs (e.g. Castel, 2007). That is, with an anticipatory design, participants could choose to adjust how they engage in retrieval upon being presented with a potential high-value reward, before encountering the probe and before initiating the attempt to retrieve. However, in the current study, participants could not selectively employ such an approach. We speculate that rewards acted on the reactivated representations of the associated images. Thus, rather than impact how participants approached the retrieval process itself, our interpretation is that rewards influenced the contents of retrieval and/or how those contents were processed after participants had made the attempt to retrieve. Surprising rewards can promote enhanced connectivity between task-specific regions, the hippocampus, and the striatum (Calderon et al., 2021). Potentially, reward presentation may strengthen reactivated representations, leading to the memory facilitation that we find for twice-presented rewards. Likewise, random reward presentation during study is thought to drive dopaminergic consolidation mechanisms (Murayama & Kitagami, 2014); the random rewards in the present study may drive similar processes following retrieval. Although future neuro-imaging work is needed to directly test the link between cortical reactivation and reward system activation, our findings are consistent with the interpretation that post-retrieval rewards may act directly on reactivated memories. Alternatively, rewards may induce a content-general increase in attention to the lingering internal representation, which may take the form of explicit rehearsal of the retrieved information. Sustained attention may extend the window of what is effectively test-phase encoding (Nyberg, Habib, & Tulving, 2000), facilitating later memory.

We find robust effects of retrieval practice vividness on subsequent memory. We replicate prior work and find that high vividness ratings during retrieval practice are associated with greater hit and false-alarm rates relative to not practiced items (Lee et al., 2018). We also find lower hit rates for items low in vividness. Our interpretation is that the consequences of retrieval practice vary depending on the strength of the retrieved information. Our findings are in line with the non-monotonic plasticity hypothesis (Poppenk & Norman, 2014), whereby moderate reactivation of an item may lead to weakening of the representation of that item (Kim, Lewis-Peacock, Norman, & Turk-Browne, 2014; Newman & Norman, 2010). Thus, it may be that low-vivid items in the present study were partially reactivated and therefore weakened by practice.

Repeated post-retrieval rewards improve subsequent memory for low-vivid items. We find that practiced items with low vividness ratings have lower subsequent hit rates than not practiced items. However, we find that subsequent memory is comparable for low-vivid practice *rewarded* items relative to not practiced items. Thus, whereas low-vivid items may be weakened through retrieval practice (Poppenk & Norman, 2014), rewards may counteract this weakening, potentially by amplifying below-threshold signals – moving the reactivated memory out of the ‘moderate’ zone and into a higher reactivation state. Such strengthening may be accomplished by attention to the reactivated memory, potentially via rehearsal processes.

It is important to note that individuals reactivate both item-specific and category-level information while making vividness judgments (e.g., Kuhl et al., 2013). In contrast to item-specific reactivation, category reactivation promotes false alarms (Lee et al., 2018). The logic is that verbatim, specific remembering (e.g., the exact image of Britney Spears) facilitates endorsement of that same image and rejection of lure images (e.g., a different image of Britney Spears; Brainerd & Reyna, 2002). In contrast, remembering gist-level detail (e.g., Britney Spears generally or more broadly, faces) will lead to erroneous endorsement of lure images. Thus, the impact that rewards have on subsequent memory should differ based on what is reactivated – item-specific vs. category-level information. To the extent that rewards strengthen reactivated content, strengthening item-specific information should facilitate later memory, whereas strengthening category-level information should impair later memory by increasing false-alarm rates. Although we cannot draw strong conclusions on the basis of null results, given the lack of an effect of rewarded retrieval practice on false alarms, we speculate that extrinsic rewards in the current study are likely reinforcing both item and category information, as both types of information contribute to vivid remembering (Lee et al., 2018). Thus, post-retrieval rewards could be both beneficial and maladaptive depending on the exact contents of retrieval.

Our use of random rewards was motivated by several factors; however, random rewards are not strongly ecologically plausible. We used random rewards so as not to link rewards to specific behaviors, contexts, and/or motor responses. Such a structure would likely motivate participants to respond ‘high vivid’ to all retrieval practice trials and/or only attend to a specific category (e.g., faces). Additionally, and perhaps most critically, our goal was to *not* induce pre-retrieval reward anticipation. Reward anticipation can influence participant strategies (Bowen, Marchesi, & Kensinger, 2020; Castel, 2007) such that non-random rewards would likely alter participants’ processing of the word cues rather than, or in addition to, processing of the reinstated image. More broadly, anticipation of a future reward may modulate how participants engage the retrieval process itself. A high-value potential future reward may motivate participants to engage in greater retrieval effort. In contrast, the use of random rewards in the present study is less likely to modulate retrieval effort or initiation and instead is more likely to modulate how participants process the retrieved information.

However, truly random rewards are unlikely in the real world, where rewards are instead linked to specific behaviors. Both positive and negative reward prediction errors (RPEs) – receiving unexpected rewards or unexpected punishments – modulate neural signals and drive behavior (Ergo, De Loof, & Verguts, 2020; Jang, Nassar, Dillon, & Frank, 2019; Rouhani & Niv, 2021; Schultz, Dayan, & Montague, 1997; Scimeca, Katzman, & Badre, 2016; Zaghloul et al., 2009). Specifically, RPEs lead to reward system responses and can either increase (following positive RPEs) or decrease (following negative RPEs) the likelihood that an organism subsequently engages in the behavior that produced the outcome. It is unclear from the present work whether reward expectancy violations at test would have a similar impact on the contents of retrieval and subsequent memory. Future work will be needed to directly test this possibility.

Extrinsic rewards can negatively impact behavior by altering arousal levels (Cheng et al., 2020) and interfering with intrinsic rewards (Deci, Koestner, & Ryan, 1999). Successful retrieval may be intrinsically rewarding (Smith & Long, 2024; Speer et al., 2014) as suggested by the finding that successful retrieval is followed by reward system activation (Spaniol et al., 2009). Such intrinsically driven signals may, similar to the present study, act on the contents of retrieval to strengthen reactivated representations. However, extrinsic rewards can diminish the impact of internal motivation (Dickerson & Adcock, 2018; Xue et al., 2023). Thus, extrinsic rewards during retrieval practice may interfere with intrinsic reward signals and inconsistently impact later memory.

Taken together, our findings demonstrate that rewarded retrieval practice facilitates subsequent recognition. In particular, low-vivid retrieval practice items – which would typically be remembered worse than not practiced items – appear to be ‘amplified’ by random extrinsic rewards. These findings suggest that the reward system may reinforce the contents of retrieval, impacting subsequent memory. Thus, in addition to modulating anticipatory strategies and potentially how individuals engage retrieval, rewards may also modulate the processes that act on what is retrieved.

## Data Availability

All study data and materials are publicly available (osf.io/y5ac8).

## Code Availability

All code is publicly available (osf.io/y5ac8).

## Declarations

### Open Practices Statement

The hypotheses, methods, and analysis plan were preregistered (https://osf.io/gebm4) prior to data collection. All pre-registered analyses were performed. Additional exploratory analyses, not preregistered, were also performed. All study data, materials, and code are publicly available (osf.io/y5ac8).
